# Impact of linkage quality on inferences drawn from analyses using data with high rates of linkage errors in rural Tanzania

**DOI:** 10.1186/s12874-018-0632-5

**Published:** 2018-12-10

**Authors:** Christopher T. Rentsch, Katie Harron, Mark Urassa, Jim Todd, Georges Reniers, Basia Zaba

**Affiliations:** 1 Department of Population Health, Faculty of Epidemiology and Population Health, London School of Hygiene & Tropical Medicine, Keppel Street, London, WC1E 7HT UK; 2 UCL GOS Institute of Child Health, London, UK; 3 The TAZAMA Project, National Institute for Medical Research, Mwanza, Tanzania; 4 MRC/Wits Rural Public Health and Health Transitions Research Unit (Agincourt), School of Public Health, Faculty of Health Sciences, University of the Witwatersrand, Johannesburg, South Africa

**Keywords:** Record linkage, Linkage error, Bias, Data accuracy, HIV, Sub-Saharan Africa

## Abstract

**Background:**

Studies based on high-quality linked data in developed countries show that even minor linkage errors, which occur when records of two different individuals are erroneously linked or when records belonging to the same individual are not linked, can impact bias and precision of subsequent analyses. We evaluated the impact of linkage quality on inferences drawn from analyses using data with substantial linkage errors in rural Tanzania.

**Methods:**

Semi-automatic point-of-contact interactive record linkage was used to establish gold standard links between community-based HIV surveillance data and medical records at clinics serving the surveillance population. Automated probabilistic record linkage was used to create analytic datasets at minimum, low, medium, and high match score thresholds. Cox proportional hazards regression models were used to compare HIV care registration rates by testing modality (sero-survey vs. clinic) in each analytic dataset. We assessed linkage quality using three approaches: quantifying linkage errors, comparing characteristics between linked and unlinked data, and evaluating bias and precision of regression estimates.

**Results:**

Between 2014 and 2017, 405 individuals with gold standard links were newly diagnosed with HIV in sero-surveys (*n* = 263) and clinics (*n* = 142). Automated probabilistic linkage correctly identified 233 individuals (positive predictive value [PPV] = 65%) at the low threshold and 95 individuals (PPV = 90%) at the high threshold. Significant differences were found between linked and unlinked records in primary exposure and outcome variables and for adjusting covariates at every threshold. As expected, differences attenuated with increasing threshold. Testing modality was significantly associated with time to registration in the gold standard data (adjusted hazard ratio [HR] 4.98 for clinic-based testing, 95% confidence interval [CI] 3.34, 7.42). Increasing false matches weakened the association (HR 2.76 at minimum match score threshold, 95% CI 1.73, 4.41). Increasing missed matches (i.e., increasing match score threshold and positive predictive value of the linkage algorithm) was strongly correlated with a reduction in the precision of coefficient estimate (R^2^ = 0.97; *p* = 0.03).

**Conclusions:**

Similar to studies with more negligible levels of linkage errors, false matches in this setting reduced the magnitude of the association; missed matches reduced precision. Adjusting for these biases could provide more robust results using data with considerable linkage errors.

## Background

A growing number of demographic and epidemiological research studies are conducted using linked datasets from multiple sources [1]. In the absence of unique identifiers, record linkage – the matching of an individual’s records between two or more data sources [2, 3] – often relies on a set of personal identifiers (e.g., names, address, date of birth) that are reported with error or are dynamic (e.g., name or residence changes). Errors arising during the linkage process because of imperfect identifiers can result in two types of linkage errors: false matches (records of two different individuals are erroneously linked) and missed matches (records belonging to the same individual are not linked). These linkage errors have been shown to impact the bias and precision of subsequent analyses [4, 5]. Even at error rates < 1%, false matches typically weaken associations between variables captured in different datasets and bias coefficients toward a null association [5] while missed matches result in a decreased analytic sample size and thus statistical power, and potentially underestimate exposures and outcomes of interest [6, 7]. Globally, there is a lack of guidance on how to measure the impact of linkage errors on analyses of linked data [8, 9]. However, the few studies that exist are predominantly conducted in settings with very low linkage errors, such as the United Kingdom, United States, and Australia [10, 11]. Whether and how analyses are affected by linkage errors in settings with lower data quality and therefore more substantial linkage errors, such as sub-Saharan Africa, remains unknown.

A recent Wellcome Trust report detailed how record linkage adds to the value of medical research in low- and middle-income countries [1]. A unique challenge exists in these settings, particularly in sub-Saharan Africa, where there is an overall lack of electronic data available for linkage and relatively poor quality of variables that could be used by a linkage algorithm. Because of this, very few record linkage projects have been undertaken throughout the region [12–15], and the absence of gold standard linked data complicate those that have used automated linkage. In a rural ward of ~ 35,000 residents in northwest Tanzania with a history of community-based HIV surveillance, we developed and implemented a novel approach to record linkage, which we term point-of-contact interactive record linkage (PIRL) [16–18]. PIRL, described later in more detail, is a semi-automatic record linkage process that incorporates human inspection of potential matches identified by a probabilistic linkage algorithm whilst in the presence of the individual whose records are being linked, which contrasts with a more conventional approach where record linkage is done automatically with no human involvement. PIRL has the advantage that uncertainty surrounding identities can be resolved during a brief interaction whereby extraneous information (e.g., household membership) can be referred to as an additional criterion to adjudicate between multiple potential matches. Largely due to the interaction with those who are the target of the linkage and the ability to perform repeated searches through the database, PIRL has been shown to outperform automated linkage for identifying matches, which have been affected by the substantial data quality issues in similar settings [18]. The gold standard linked database created by PIRL allows for the first known attempt to evaluate the impact of linkage errors on subsequent analyses in a setting with substantial linkage errors.

The linked data infrastructure created by PIRL includes gold standard links between HIV serological survey data and manually digitised medical records from three clinics serving the surveillance population, two of which offer HIV testing services while the third enrols HIV-positive individuals into care. As an illustrative example to evaluate linkage errors, we tested whether individuals who receive their first HIV diagnosis during a village-based HIV serological survey enrol for HIV care services quicker than those who receive their first HIV diagnosis in a clinic that also offers HIV testing. For this analysis, we first assessed the relationship between diagnosis location and time to enrolment into HIV care in the gold standard linked data. We then conducted automated record linkage, a process that included no human interaction or involvement like PIRL, to create four test datasets based on varying levels of match score threshold. Linkage errors, including false and missed matches, were quantified in each test dataset, overall and by individual characteristics. Finally, we determined whether and how linkage errors impacted the analysis of the primary research question by comparing the characteristics of linked and unlinked records and the bias and precision of regression coefficients.

## Methods

### Data sources

The Kisesa observational HIV cohort study was established in 1994 and is located in a rural ward in the Magu district of Mwanza region in northwest Tanzania [19]. The study includes multiple rounds of health and demographic surveillance system (HDSS) surveys that cover the entire population of ~ 35,000 residents, and multiple rounds of population-based HIV sero-surveys, in which adults aged 15 years or older living in the Kisesa HDSS study area are invited to attend temporary village-based clinics for a personal interview and HIV test. A government-run health centre serving the HDSS population includes an HIV testing and counselling clinic (HTC), an antenatal clinic (ANC) offering HIV testing, and an HIV care and treatment centre (CTC). For the HTC and ANC, we developed electronic databases and digitised the paper-based logbooks using a double-entry system where two different fieldworkers independently capture each book, and any discrepancy between fields were reconciled in a third cleaning stage. The CTC databases have been fully digitised, and data clerks regularly update and run data checks on these data. Ethical approval was obtained from the National Institute for Medical Research, Tanzania (reference no. NIMR/HQ/R.8c/Vol.II/436 and MR/53/100/450), and the London School of Hygiene and Tropical Medicine (Project ID #8852). Informed written consent (including consent to link data sources in the PIRL study) was obtained from all participants. Parental written consent was additionally obtained for participants < 18 years of age.

### Linkage

Participants’ records from all sero-survey rounds were cross-referenced with their HDSS identifiers as part of the identification process during the survey interview. Records from the three clinics were linked to the HDSS database using PIRL, which has been described elsewhere [17, 18]. Briefly, as individuals arrived to any of the three clinics and consented to be in the study, fieldworkers entered their personal and residence details into specialised computer software [16], which used a probabilistic linkage algorithm to search the HDSS database. The algorithm used to search for possible matches was based on the Fellegi-Sunter record linkage model [2, 3], and incorporated the following data fields: up to three names for the individual; sex; year, month, and day of birth; village and sub-village; up to three names of a household member; and up to three names for the ten-cell leader of the patient. A ten-cell leader is an individual who acted as a leader for a group of ten households and these positions have been relatively stable over time. While searching through potential matches, the fieldworker could view the full list of household members associated with each HDSS record as an additional step to adjudicate true matches. The fieldworker then interacted with the patient to identify which HDSS record(s), if any, were a true match.

Multiple data checks were performed within the software and on the back-end database to ensure the links made with PIRL were true matches. First, the software displayed warning messages to the fieldworkers if they attempted to match to a record that had an absolute difference in birth year of > 10 years, or the entered names did not agree with the names listed on the selected HDSS record as measured by a Jaro-Winkler string comparator [20]. The linkage algorithm allowed for all pairwise comparisons between listed names on clinic and HDSS records because the order of names is relaxed in this setting and HDSS records only hold up to two names while other data sources often store more than two names. Further, the lead author performed periodic and manual, back-end inspection of the data to verify the matches made in the field. These data integrity checks flagged individuals who were matched to multiple HDSS records with large age differences (> 10 years), of conflicting sex, within the same household, or with overlapping household residency episodes in which one record’s start date occurred before another record’s end date. Over the study period, eight PIRL matches were deemed unlikely and deleted.

Using links made during the sero-survey and PIRL as the gold standard, we performed automated probabilistic record linkage using the same algorithm used in the PIRL software but limited to identifiers collected in the sero-survey and clinic databases. Automated record linkage has been well described [21–25]. Briefly, a match score (i.e., the weighted likelihood a record-pair is a link or non-link) was calculated for all pairwise comparisons between the patient registry and the HDSS database. The HDSS record with the highest match score was selected for each record in the patient registry. When performing automated linkage, a match score threshold is selected to determine what constitutes a link versus a non-link. The placement of the threshold can be a matter of trial and error [26]. Additionally, a match score is not a standardised metric and can be greatly influenced by the number of identifiers used in the linkage algorithm. To show how the impact of linkage errors on subsequent analyses were affected by the placement of the match score threshold, we created separate analytic test datasets at various thresholds based on percentiles of the distribution of match scores, rather than absolute scores, among true matches. By selecting thresholds based on percentiles and not absolute scores, our findings may be more generalizable to other settings. In this sample, match scores among true matches ranged from − 21 to 61. We created four analytic datasets based on the following thresholds: (a) all matches above the minimum match score (threshold = − 21), (b) 25th percentile (low threshold = 13), (c) 50th percentile (medium threshold = 24), and (d) 75th percentile (high threshold = 35). Higher thresholds represent more conservative definitions on what constituted a true match. In cases where matches were missed (i.e. the match score for a true record-pair fell below a particular threshold), these data were not included in the analytic dataset, thereby reducing the sample size of the dataset. The PIRL links made between the CTC and HDSS databases were then used for the entire sample to identify those who registered for HIV care.

### Analytic sample

We included all individuals with a gold standard link who received their first positive HIV diagnosis in the sero-survey, HTC, or ANC between December 2014 and October 2017. Individuals were excluded if they were younger than 15 years (to be consistent with the 15-year age limit in the sero-survey), had evidence of a previous positive HIV diagnostic test or registered for HIV care prior to their HIV test (repeat testers), or reported residence outside the HDSS area or were not seen in the 2016/17 HDSS round (non-residents). Repeat testers and non-residents were excluded because these groups are likely to achieve the outcome (registered for HIV care) at different rates than individuals newly diagnosed with HIV and residents. We extracted demographic and spatial characteristics including sex, age, rurality of sub-village (rural, peri-urban, or urban), whether the sub-village of residence had a paved road, and geodesic distance between an individual’s household and the CTC.

### Statistical analyses

Chi-square and Fisher’s exact tests were used to assess differences between individuals who were diagnosed with HIV by testing modality, i.e. in the community-based sero-survey versus walk-in clinic (either HTC or ANC) during the study period in the gold standard data. At each match score threshold, we classified links made by the automated linkage as true, false, or missed matches and compared characteristics between these groups using standardised differences [27]. Standardised differences of 0.2, 0.5, and 0.8 represented small, moderate, and large standardised differences, respectively, comparing true matches with false and missed matches [28]. Cox proportional hazards regression models were used to compare HIV care registration rates by testing modality (sero-survey vs. clinic) in each dataset created by the automated linkage. Individuals were censored at first CTC visit, death, or 90 days after positive HIV diagnosis. Models were adjusted for age, sex, rurality of sub-village, whether the sub-village had a paved road, and distance to the CTC. We evaluated for bias in precision by comparing regression coefficients and standard errors of the primary exposure variable (testing modality) in the gold standard data with those obtained at each selected match score threshold. Statistical analyses were performed using SAS version 9.4 (SAS Institute Inc., Cary, NC, USA).

## Results

### Gold standard links

During the study period, 263 and 142 individuals with gold standard links received their first positive HIV diagnosis in the sero-survey and clinics, respectively (total *n* = 405). Among clinic patients, 126 (89%) HIV diagnoses occurred in the HTC and the remaining 16 (11%) diagnoses were made in the ANC. Participants diagnosed in the sero-survey were more likely to be older, from more rural areas, and reside further from the CTC than those who were diagnosed in a clinic (all *p* < 0.02) (Table 1). Over half (*n* = 75 [53%]) of individuals diagnosed in a clinic subsequently registered for HIV care by the study cut-off date, compared to 42 (16%) of those diagnosed in the sero-survey (*p* < 0.0001).

**Table 1:** Characteristics of patients in the analytic sample

Characteristic	Sero-survey participants (*n* = 263)	Clinic patients (*n* = 142)	*p*-value
Clinic			
ANC	–	16 (11.3)	–
HTC	–	126 (88.7)	
Sex			
Female	173 (65.8)	98 (69.0)	0.5092
Male	90 (34.2)	44 (31.0)	
Age, years			
15–29	62 (23.6)	51 (35.9)	0.0222
30–39	96 (36.5)	53 (37.3)	
40–49	59 (22.4)	22 (15.5)	
50+	46 (17.5)	16 (11.3)	
Village			
Igekemaja	27 (10.3)	14 (9.9)	0.0167
Ihayabuyaga	30 (11.4)	6 (4.2)	
Isangijo	27 (10.3)	14 (9.9)	
Kanyama	38 (14.5)	23 (16.2)	
Kisesa	73 (27.8)	51 (35.9)	
Kitumba	32 (12.2)	26 (18.3)	
Welamasonga	36 (13.7)	8 (5.6)	
Rurality of sub-village			
Rural	140 (53.2)	55 (38.7)	0.0204
Peri-urban	54 (20.5)	39 (27.5)	
Urban	69 (26.2)	48 (33.8)	
Sub-village had paved road			
Yes	109 (41.4)	70 (49.3)	0.1290
No	154 (58.6)	72 (50.7)	
Distance from household to CTC, km			
< 1	53 (20.2)	37 (26.1)	0.0162
1–1.9	58 (22.1)	45 (31.7)	
2–4.9	60 (22.8)	29 (20.4)	
5–11	92 (35.0)	31 (21.8)	
Registered at CTC	42 (16.0)	75 (52.8)	< 0.0001

Abbreviations: CTC - HIV care and treatment centre; ANC - antenatal clinic; HTC - HIV testing and counselling clinic

Note: all statistics are given in n (%); differences tested using chi-square

### Automated linkage

Most identifiers used by the linkage algorithm were complete or nearly complete in the sero-survey and clinic databases, including two names, year of birth, sex, village, and sub-village information (all ≥99.3% complete) (Table 2). A majority (72%) of sero-survey records also included two names of another household member, 48% included two names of the household’s ten-cell leader, and 13% had a third name for the individual. Most (89%) clinic records held information on a third name for the individual, > 75% up to two names of the household’s ten-cell leader, and 12% included two names for another household member. The HDSS database had high levels of completeness (all > 99%) on all identifiers used by the linkage algorithm except for a third name, which is not collected in the HDSS system.

**Table 2:** Completeness of matching identifiers in clinic data and demographic surveillance data

	% records with complete information		
Matching identifier	Sero-surveys (*n* = 263)	Clinic data (*n* = 142)	HDSS data (*n* = 99,866)
First name	100.0%	100.0%	100.0%
Second name	100.0%	100.0%	100.0%
Third name	13.3%	88.7%	–
Year of birth	100.0%	100.0%	99.4%
Sex	100.0%	100.0%	100.0%
Village	100.0%	99.3%	100.0%
Sub-village	100.0%	99.3%	100.0%
TCL first name	48.3%	91.5%	99.4%
TCL second name	48.3%	74.6%	99.4%
Household member first name	71.5%	11.3%	99.9%
Household member second name	71.5%	11.3%	99.9%

Abbreviations: HDSS - health and demographic surveillance system; TCL - ten-cell leader

**Fig. 1:**
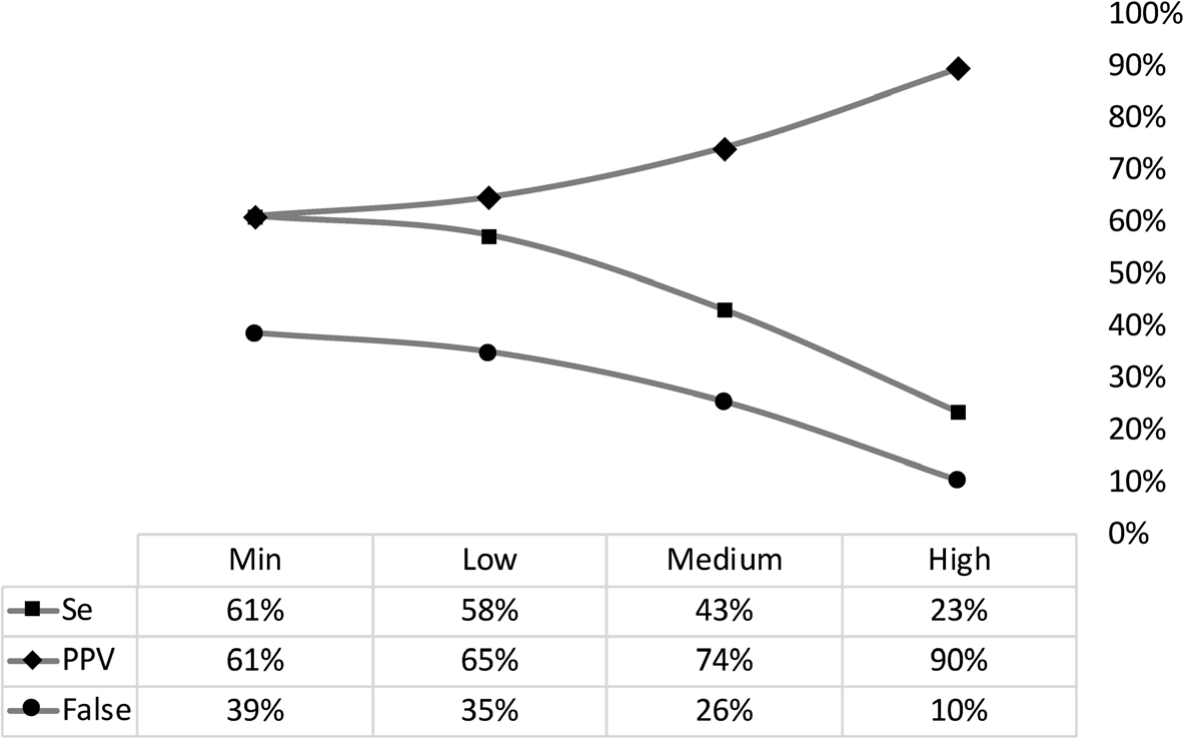
Sensitivity (Se), positive predictive value (PPV), and false match rate (False), by match score threshold. *Notes:* Se = linked records over N true matches; PPV = true matches over N linked records; False = false matches over N linked records; or simply the inverse of PPV

Of the 405 gold standard links, automated linkage correctly identified 248 records, falsely matched 157 records, and missed 157 records at the minimum match score threshold. This resulted in a sensitivity and positive predictive value (PPV) of 61% and a false match rate of 39% (Fig. 1). At the high match score threshold, automated linkage correctly identified 95 records, falsely matched 11 records, and missed 310 records, which equated to a sensitivity of 23%, PPV of 90%, and false match rate of 10%.

### Linked sample characteristics

The frequency of the primary exposure variable, the location in which an individual received their first positive HIV diagnostic test, differed between true, false, and missed matches at all match score thresholds Table 3. Compared to linked true matches, false and missed matches were more likely to receive their HIV-positive test in a clinic than the sero-survey. Increasing the threshold minimised but did not eliminate the differences between true matches and false matches.

**Table 3:** Comparison of regression model diagnostics by match score threshold

Sample	n	β	SE	χ^2^	p	HR (95% CI)	PPV
Gold standard	405	1.61	0.2033	62.4	<.0001	4.98 (3.34, 7.42)	–
Probabilistic linkage threshold, by match score threshold							
minimum	405	1.02	0.2383	18.2	<.0001	2.76 (1.73, 4.41)	0.612
low	359	1.20	0.2579	21.7	<.0001	3.32 (2.00, 5.51)	0.649
medium	235	0.86	0.4621	3.5	0.0615	2.37 (0.96, 5.87)	0.745
high	106	0.53	1.1707	0.2	0.6501	1.70 (0.17, 16.87)	0.896

Abbreviations: n - sample size; β - primary exposure coefficient; SE - standard error; χ^2^ - chi-square; p - p-value; HR - hazard ratio; CI - confidence interval; PPV - automated linkage algorithm’s positive predictive value

Note: All models adjusted for age, sex, sub-village, and distance from household to CTC

The frequency of the outcome variable, registering at the CTC, also differed significantly between true matches and false matches, particularly at lower match score thresholds. Compared to linked true matches, false matches were less likely to have registered at the CTC at every match score threshold except for the high threshold.

There were also differences between true, false, and missed matches with respect to variables used as adjusting factors. False matches were more likely to be younger, from more rural areas, and reside at greater distances from the CTC. There were minimal differences between true and false matches by sex in analytic samples created using lower match score thresholds; however, false matches were more likely than true matches to be male at the medium and high match score thresholds.

### Modelled estimates

**Fig. 2:**
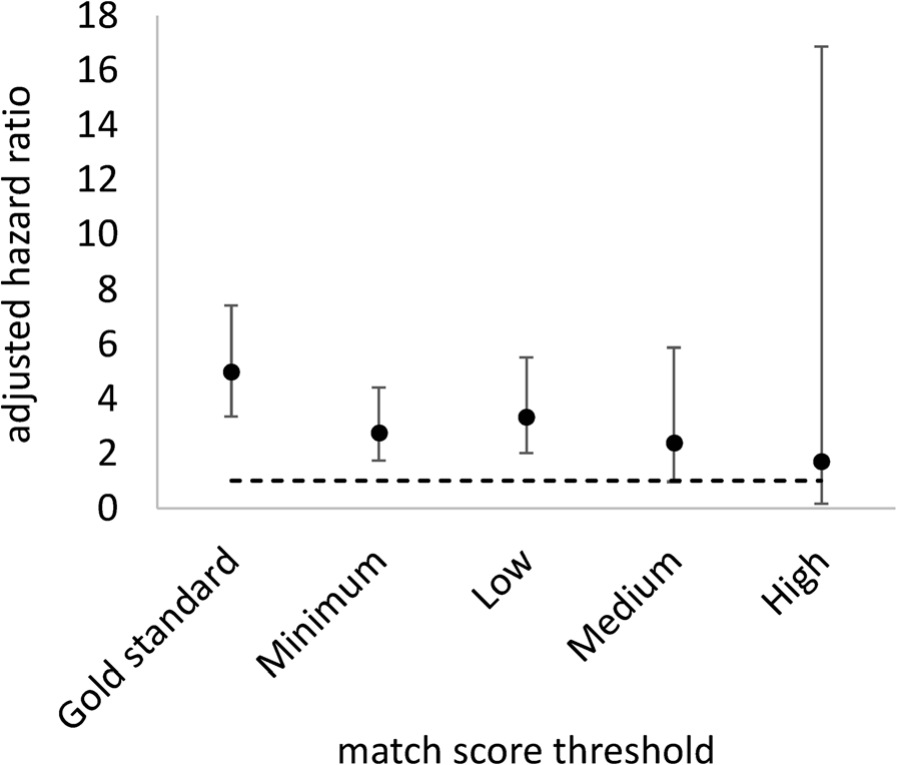
Associations between primary exposure and outcome variables by match score threshold

There was a significant association between testing modality and time to registration at the CTC in the linked gold standard data in favour of those receiving their diagnosis at a walk-in clinic (adjusted hazard ratio [HR] 4.98, 95% confidence interval [CI] 3.34, 7.42) (Table 3). Bias was present at each match score threshold in the automated linked datasets. The significant positive association was still found, though much attenuated, at the minimum threshold (HR 2.76, 95% CI 1.73, 4.41) and low threshold (HR 3.32, 95% CI 2.00, 5.51) (Fig. 2). The association was not found at the medium threshold (HR 2.37, 95% CI 0.96, 5.87) nor the high threshold (HR 1.70, 95% CI 0.17, 16.87). An increase in the number of missed matches from the analytic dataset (i.e. increasing the match score threshold and positive predictive value of the linkage algorithm) was strongly correlated with a reduction in the precision of the primary exposure coefficient (R^2^ = 0.97; *p* = 0.03).

## Discussion

This paper provides original evidence that bias and precision in analyses using linked data are impacted by substantial linkage errors similarly to how they are impacted by more negligible linkage errors. With the recent availability of gold standard linked data in this East African setting, we asked a timely research question and assessed how our conclusions would have changed if instead of using gold standard linked data, we used automated record linkage, a less resource-intensive but less accurate form of record linkage. We evaluated the quality of automated linkage and identified potential sources of bias by quantifying false and missed matches, comparing characteristics between linked and unlinked data, and comparing regression coefficients at various match score thresholds in sensitivity analyses. High levels of linkage errors in this setting (e.g. false match rates up to 40% observed at the minimum threshold) introduced bias at all match score thresholds. False matches reduced the magnitude of the association between the tested exposure and outcome while increasing numbers of missed matches reduced the precision of these estimates, which is comparable to analyses in settings with higher quality data [5–7, 11, 29].

We used standardised differences to identify variables that were more affected by linkage error and potential sources of bias as was done in previous studies [30]. We found strong evidence of selection bias based on who was included in the analytic datasets since frequencies of the primary exposure, outcome, and some adjusting variables differed significantly between true, false, and missed matches at all match score thresholds. Increasing the match score threshold attenuated differences between true and false matches but also exacerbated differences between true and missed matches. The trade-off between false and missed matches when comparing characteristics between linked and unlinked data has also been found in other settings with low levels of linkage errors [31].

We found measurable bias in the regression coefficient of the primary exposure at every match score threshold. Selection bias is likely to have impacted the analyses given that selection into the linked datasets was related to both the exposure and outcome [32, 33]. Therefore, conditioning or limiting the analyses to records that were linked could therefore induce a protective relationship between the exposure and outcome, as we found in this analysis. One method to potentially correct for this bias is to use multiple imputation to handle missing values due to unlinked records [29], which could employ the match weights from the linkage procedure to inform priors during the imputation process [34, 35]. We also found that the number of missed matches increased at higher thresholds which resulted in a decreased analytic sample size and thus statistical power as evidenced by larger standard errors and wider confidence intervals compared to lower thresholds. Our identification of bias towards a null association with gains in precision at these lower thresholds is substantiated by previous research that showed similar trends in settings with minimal linkage errors [5, 29].

Our findings suggested the optimal point that balanced trade-offs between false and missed matches (i.e., PPV and sensitivity) was at the minimum match score threshold. However, optimisation in this context is inappropriate because there were large biases in the primary regression coefficient at this and all other match score thresholds, which would have led to misleading interpretations of the results. While the consequence of optimisation in our primary analysis would have resulted in a biased measure of the association between variables found in different data sources, a potentially more appropriate use of optimisation may have been to obtain a count outcome from a single data source. The proportion of individuals who registered at the CTC (outcome) was 29% in the gold standard data and ranged between 17 and 19% in the automated linked datasets. Therefore, if our research question was to obtain the proportion or rate of individuals who registered at the CTC our conclusions would also have been meaningfully different at every match score threshold. While optimisation was possible with our data, we conclude there was no optimal threshold that balanced trade-offs between PPV and sensitivity as well as resulted in unbiased associations between two variables or count outcomes of a single variable.

A strength of this analysis was the access to individual-level data collected in the PIRL software, clinics, and sero-surveys. This information is often only available to individuals performing the linkage and not to researchers conducting analyses [36–39] and allowed us to have full control of the automated linkage process including data pre-processing to improve the quality of the variables used in the algorithm. Most of the identifiers used by the automated linkage algorithm had no or very little missing data, including names, year of birth, sex, village and sub-village. While the algorithm embedded in the PIRL software utilised a larger set of personal identifiers, this restricted set of variables has been shown to drive the success of the linkage algorithm in our PIRL software [18].

There were some limitations. First, the magnitude of the tested association between the selected exposure and outcome was large in the gold standard data, which was probably why the conclusions of the primary regression analysis were similar in the automated linked datasets at the lower match score thresholds even after measurable attenuation in the estimate. It is likely that a more modest association found in the gold standard data would have resulted in a null association and therefore different conclusions as has been found in other studies [31]. Second, the relatively small sample size in the gold standard data did not allow us to assess linkage bias at match score thresholds higher than the 75th percentile.

## Conclusions

Recently, there has been increased attention on how errors arising during the linkage process impacts inferences drawn from analyses using imperfectly matched data, but predominately in high-income countries with negligible linkage errors. We provided original evidence that the impact of linkage quality is similar in a low-income country setting with substantial linkage errors. We plan to investigate methods that minimise or correct for these biases and provide more robust results using data with considerable linkage errors. Until these analyses are complete, our results suggest that researchers in similar settings desiring to perform probabilistic record linkage should allocate resources toward PIRL or similar system[s].

## Abbreviations

ANC: Antenatal clinic
CI: Confidence interval
CTC: HIV care and treatment centre
HDSS: Health and demographic surveillance system
HIV: Human immunodeficiency virus
HR: Hazard ratio
HTC: HIV testing and counselling clinic
PIRL: Point-of-contact interactive record linkage
PPV: Positive predictive value
